# Rapid-onset postoperative acute kidney injury is associated with mortality in patients with postcardiotomy cardiogenic shock

**DOI:** 10.3389/fcvm.2025.1580599

**Published:** 2025-06-13

**Authors:** Naoki Tadokoro, Keita Saku, Kohei Tonai, Yuki Tadokoro, Reiko Kutsuzawa, Satsuki Fukushima

**Affiliations:** ^1^ Department of Cardiac Surgery, National Cerebral and Cardiovascular Center, Suita, Osaka, Japan; ^2^ Department of Cardiovascular Dynamics, National Cerebral and Cardiovascular Center Research Institute, Suita, Osaka, Japan

**Keywords:** postcardiotomy cardiogenic shock, acute kidney injury, mechanical circulatory support, veno-arterial extracorporeal membrane oxygenation, extracorporeal life support, ventricular assist device

## Abstract

**Background:**

Post-cardiotomy cardiogenic shock (PCCS) is a serious condition that necessitates veno-arterial extracorporeal membrane oxygenation (VA-ECMO). Although acute kidney injury (AKI) often complicates PCCS, its specific effects on patient outcomes remain unclear. This study seeks to evaluate the impact of AKI on 90-day mortality.

**Methods:**

This retrospective study included 91 patients with postoperative cardiogenic shock requiring venoarterial extracorporeal membrane oxygenation following cardiac surgery between 2013 and 2023. Rapid-onset AKI was defined as KDIGO Stage 2 or higher within 24 h of ICU admission. Survival was analyzed using Kaplan–Meier and Cox regression methods to assess its association with 90-day mortality.

**Results:**

Twenty-four patients (26.4%) were classified as rapid-onset AKI. The median age, primary diagnosis, and preoperative serum creatinine levels were similar between groups. However, the rapid-onset AKI group had a preoperative lower left ventricular ejection fraction (42.5% vs. 60.0%, *p* = 0.006), longer cardiopulmonary bypass time (332 vs. 245 min, *p* = 0.009), and a longer duration of mechanical circulatory support (6.0 vs. 2.0 days, *p* = 0.001). The success rate of weaning from mechanical circulatory support was lower (61.1% vs. 93.3%, *p* = 0.002), and the 90-day cumulative survival probability was lower in the rapid-onset AKI group (29.1% [95% confidence interval (CI): 15.6–54.4 vs. 79.1% [95% CI: 69.9–89.4], *p* < 0.001). Cox regression analysis confirmed an independent association between rapid-onset AKI and 90-day mortality (adjusted hazard ratio: 3.15, 95% CI: 1.38–7.19, *p* = 0.006).

**Conclusion:**

Rapid-onset AKI was significantly associated with increased 90-day mortality in patients with PCCS who required V-A ECMO.

## 1 Introduction

Postoperative cardiogenic shock (PCCS) is characterized as a low cardiac output syndrome accompanied by impaired peripheral perfusion. Although its incidence is relatively low, ranging from 0.5% to 1.5%, the in-hospital mortality rate remains extremely high, between 60% and 80% (1). Mechanical circulatory support devices, such as veno-arterial extracorporeal membrane oxygenation (V-A ECMO), are commonly employed in its management (2–4). Circulatory failure has been identified as a critical determinant of survival outcomes, underscoring the importance of prompt hemodynamic stabilization.

Another critical complication is Cardiac surgery-associated acute kidney injury (CSA-AKI), which is also one of the most significant postoperative complications affecting patient prognosis (5, 6). It typically arises from renal hypoperfusion and systemic inflammatory responses triggered during and after surgery (7, 8). Both PCCS and AKI are severe postoperative complications, and they can exacerbate each other's pathophysiology. Renal hypoperfusion caused by PCCS can precipitate AKI, while the progression of AKI can further worsen circulatory status through fluid overload and inflammation (9, 10).

Among these, the development of Stage 2 or higher AKI within 24 h after surgery is likely to reflect acute hemodynamic and inflammatory changes and may serve as an early warning sign of systemic deterioration. This is based on evidence indicating that the severity of renal tubular injury strongly reflects the degree of inflammatory response, and that early recognition of AKI may be useful in predicting the prognosis of CSA-AKI (11–13).

In patients with PCCS, who are already at high risk for profound circulatory instability and multi-organ dysfunction, the prognostic implications of early AKI may be particularly significant. Therefore, identifying whether AKI occurs within the first 24 h following surgery and clarifying its impact on subsequent survival is a critical clinical issue.

This study focuses on the occurrence of AKI within 24 h after cardiac surgery in patients with PCCS and investigates its association with 90-day postoperative survival. By elucidating the prognostic significance of perioperative AKI, the aim is to provide insights that may contribute to risk stratification and early therapeutic decision-making in patients with PCCS.

## 2 Materials and methods

### 2.1 Study design, cohort, and data collection

**Figure 1:**
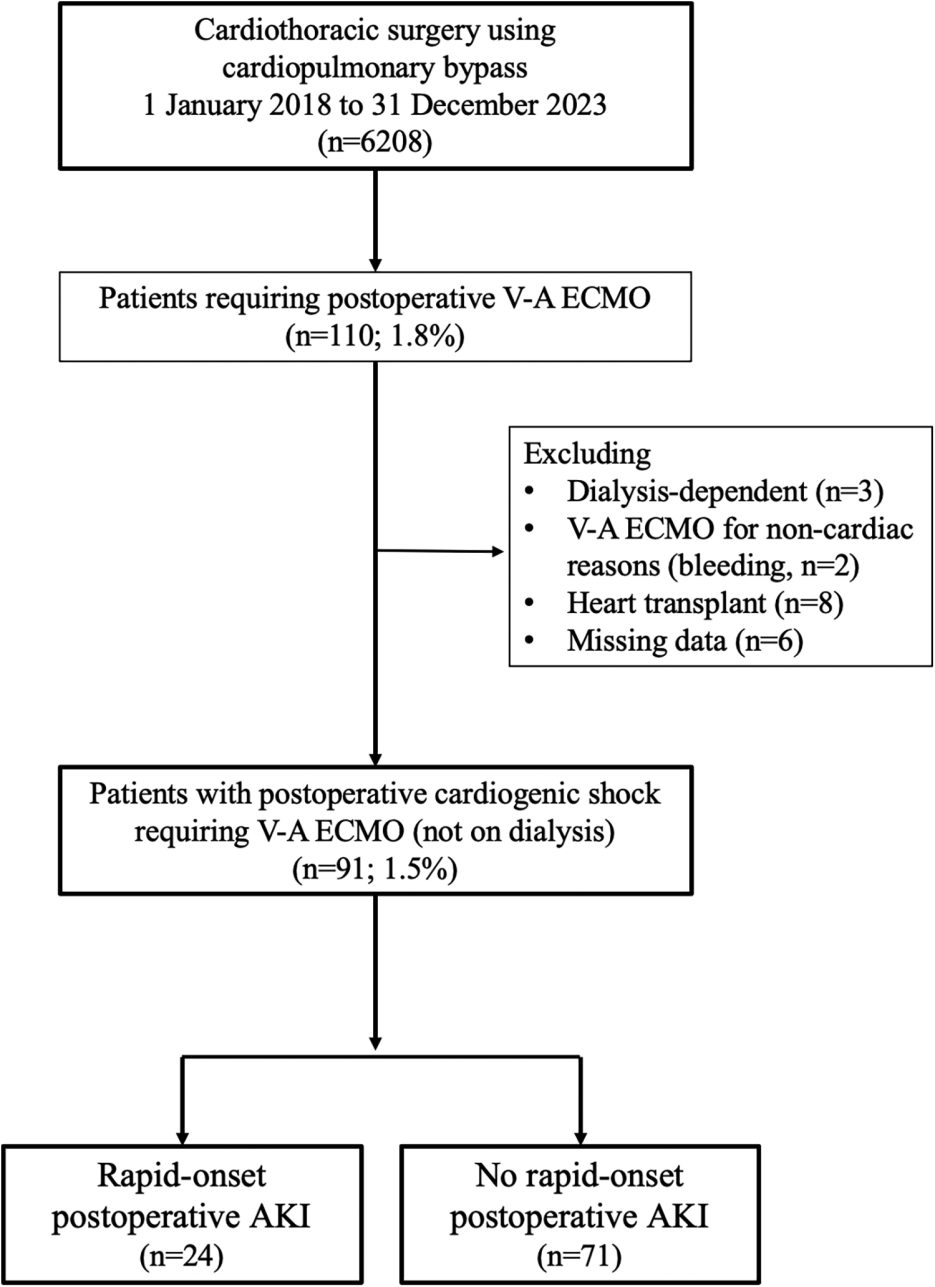
Study population and inclusion criteria for postoperative cardiogenic shock requiring veno-arterial extracorporeal membrane oxygenation.

This study was a single-center retrospective analysis conducted between January 2013 and December 2023, with data collection carried out in October 2024. A total of 6,208 patients underwent cardiothoracic surgery using cardiopulmonary bypass. 110 patients required postoperative V-A ECMO. After excluding patients who were dialysis-dependent preoperatively (*n* = 3), received V-A ECMO for non-cardiac indications (*n* = 2), underwent heart transplantation (*n* = 8), or had missing postoperative clinical data (*n* = 2), 95 patients remained for analysis as those experiencing postoperative cardiogenic shock requiring V-A ECMO (Figure 1). No major changes were made to the overall surgical techniques employed at our institution during the study period. The basic composition of the cardioplegia solution was consistently extracellular type, mixed with blood at a ratio of either 1:1 or 3:1. Cardioplegia was administered under hypothermia at 14°C for aortic procedures, while tepid cardioplegia at 29°C was used for other cases during the study period. AKI was defined according to the KDIGO criteria.

Previous studies on mortality risk and acute kidney injury (AKI) in patients supported with V-A ECMO have demonstrated that the risk is particularly elevated in those with KDIGO Stage 2 or higher (14). Therefore, to specifically evaluate the impact of hyperacute kidney injury in this study, we defined patients who met the criteria for KDIGO Stage 2 or higher within 24 h after ICU admission—either a serum creatinine level more than twice the preoperative value or urine output less than 0.5 ml/kg/h for more than 12 h—as the rapid-onset AKI group (15). Based on these criteria, 24 patients developed rapid-onset AKI, while the remaining 71 patients did not.

The primary outcome was mortality from all causes within 90 days post-surgery. Secondary outcomes included the duration and successful weaning from mechanical circulatory support.

### 2.2 Criteria and hemodynamic assessment for V-A ECMO initiation in PCCS patients

The criteria for initiating V-A ECMO in PCCS patients included an inability to wean from cardiopulmonary bypass or a cardiac index of less than 2.2 L/min/m^2^, as well as a systolic blood pressure below 90 mmHg (or a mean arterial pressure below 60 mmHg), despite optimal treatment with inotropic or vasopressor agents and/or intra-aortic balloon pumping (IABP) (2, 16). Heart failure was diagnosed based on Swan-Ganz catheter data obtained immediately before V-A ECMO initiation and transesophageal echocardiography (TEE) performed by anesthesiologists. Left ventricular failure occurs when the pulmonary artery wedge pressure (PAWP) exceeds 18 mmHg, and the ratio of right atrial pressure to pulmonary capillary wedge pressure (RAP/PCWP) falls below 0.63, or when TEE confirms preserved right ventricular function alongside a reduction in RV fractional area change (RVFAC) of less than 20%. Right ventricular failure is diagnosed when inhaled nitric oxide is administered, PAWP remains below 18 mmHg, and TEE indicates an RVFAC reduction of 20% or more. Biventricular failure is identified when PAWP exceeds 18 mmHg, the RAP/PCWP ratio is greater than 0.63, and TEE reveals an RVFAC reduction of less than 20%.

### 2.3 Mechanical circulatory system selection

In ECMO management, the choice between peripheral and central cannulation, as well as the decision to introduce LV venting, was made intraoperatively through discussions between the surgeons and anesthesiologists. The Heart Team determined the postoperative selection of mechanical circulatory support modalities, which included cardiologists, surgeons, and other relevant specialists. Before the Impella (Abiomed, Danvers, MA, USA) device was introduced in Japan in 2018, left ventricular unloading was achieved using either IABP or left atrial/ventricular venting. In Japan, upgrading to an extracorporeal ventricular assist device (Ex-VAD) was generally indicated for patients under 65, while those aged 65 years or older continued to be managed with V-A ECMO. This study was conducted at a high-volume tertiary referral center capable of advanced cardiac surgery, including heart transplantation and the implantation of durable left ventricular assist device (LVAD), where approximately 1,000 adult cardiothoracic surgeries are performed annually.

### 2.4 Hemodynamic and AKI management

In our institution, hemodynamic management was guided by cardiac function in conjunction with specific targets, including a mean arterial pressure (MAP) above 60 mmHg, arterial oxygen saturation above 90%, and venous oxygen saturation above 60% (17).

In cases where CSA-AKI was identified, efforts were made to correct hypotension and low cardiac output, and to optimize fluid balance (10, 18). Loop diuretics were the first-line treatment used to prevent or manage fluid overload in patients with AKI; however, when diuretic response was poor and rapid correction of fluid overload was necessary, renal replacement therapy (RRT) was initiated (19).

### 2.5 Weaning protocols for MCS

The decannulation process from V-A ECMO was guided by hemodynamic stability, defined as a MAP >60 mmHg with minimal inotropic support, V-A ECMO flow <3 L/min, and sustained arterial pulsatility with a pulse pressure >20 mmHg for more than 24 h (20, 21). V-A ECMO flow was reduced in 1 L/min increments with 10-minute assessments at each level, down to a minimum of 0.5 L/min. Final evaluation at 0.5 L/min focused on cardiac function. LVAD weaning followed modified Berlin criteria, including a left ventricular ejection fraction (LVEF) >45% and left ventricular end-diastolic diameter (LVDd) <55 mm; however, elective explantation was considered in selected patients with LVEF >30% and no hemodynamic deterioration during the pump-off test (22).

### 2.6 Statistical analysis

Descriptive statistics were reported as the median [interquartile range] for continuous variables, as normality was not confirmed through histograms. Categorical variables were presented as counts (percentages). The Kruskal–Wallis test was applied to compare continuous variables between groups, while Fisher's exact test was employed for categorical variables. An unadjusted survival analysis comparing the two groups based on the occurrence of rapid-onset AKI was conducted using the Kaplan–Meier method. A multivariable Cox regression analysis was conducted to explore the association between rapid-onset AKI and 90-day survival. Factors included in the Cox model were determined based on previous studies. The factors included in the Cox models are as follows: age (per 10-year increase), primary diagnosis of aortic disease, lactate levels at 24 h after ICU admission, CPB duration (per hour increase), and postoperative LVEF (per 10-% increase) (1–3, 7, 23, 24). The hazard ratio (HR) is presented with 95% confidence intervals (95% CIs). Statistical analyses were conducted at a two-sided 5% significance level. Although this study had a small sample size, a multivariable Cox analysis was conducted while acknowledging the potential risks of multicollinearity and overfitting. All statistical analyses were performed using R version 4.4.2 (The R Foundation for Statistical Computing, Vienna, Austria).

## 3 Result

### 3.1 Patients' characteristics

The median age of the patients was 71.0 years [IQR, 57.0–79.0], including 55 males (60.4%) (Table 1). The preoperative left ventricular ejection fraction (LVEF) was 59.0% [IQR, 40.0–65.0], with 19 patients (20.9%) exhibiting an LVEF of less than 35%. A history of cardiac surgery was reported in 28 patients (30.8%), while 34 patients (37.4%) underwent emergency surgery. The primary indications for surgery included valvular disease in 30 patients (33.0%), aortic aneurysm in 15 patients (16.5%), aortic dissection in 13 patients (14.3%) and ischemic heart disease in 26 patients (28.6%). The European System for Cardiac Operative Risk Evaluation (EuroSCORE) II was 11.6 [IQR, 4.9–25.7].

**Table 1:** Patient characteristics and presentation.

Variable	Category	Overall	Rapid-onset postoperative AKI	No rapid-onset postoperative AKI	*P* value
*n*		91	24	67	
Age, years		71.0 [57.0, 79.0]	74.0 [62.5, 80.2]	71.0 [57.0, 78.5]	0.430
Age > 65 years		58 (63.7)	42 (62.7)	16 (66.7)	0.920
Gender					0.145
	Male	55 (60.4)	18 (75.0)	37 (55.2)	
	Female	36 (39.6)	6 (25.0)	30 (44.8)	
Body surface area, m^2^		1.67 [1.50, 1.79]	1.67 [1.53, 1.79]	1.67 [1.50, 1.79]	0.811
Hypertension		49 (53.8)	16 (66.7)	33 (49.3)	0.219
Hyperlipidemia		29 (31.9)	10 (41.7)	19 (28.4)	0.344
Insulin-dependent diabetes		6 (6.6)	2 (8.3)	4 (6.0)	1.000
Past Smoker		28 (30.8)	7 (29.2)	21 (31.3)	1.000
Chronic lung disease		11 (12.1)	3 (12.5)	8 (11.9)	1.000
Preoperative Creatinine, mg/dl		0.97 [0.79, 1.29]	1.08 [0.81, 1.39]	0.95 [0.78, 1.21]	0.222
Estimated Glomerular Filtration Rate, ml/min/1.73 m^2^		49.0 [34.7, 65.0]	44.0 [29.3, 63.2]	50.0 [37.5, 65.2]	0.242
Recent myocardial infarction		22 (24.2)	3 (12.5)	19 (28.4)	0.201
LVEF, %		59.0 [40.0, 65.0]	42.5 [28.8, 58.5]	60.0 [45.0, 65.0]	0.006
LVEF < 30%		19 (20.9)	9 (37.5)	10 (14.9)	0.041
NYHA class					0.785
	Class I	15 (16.5)	5 (20.8)	10 (14.9)	
	Class II	33 (36.3)	8 (33.3)	25 (37.3)	
	Class III	23 (25.3)	7 (29.2)	16 (23.9)	
	Class IV	20 (22.0)	4 (16.7)	16 (23.9)	
History of Previous Cardiac Surgery		28 (30.8)	11 (45.8)	17 (25.4)	0.108
Urgency					0.405
	Elective	48 (52.7)	11 (45.8)	37 (55.2)	
	Urgent	9 (9.9)	4 (16.7)	5 (7.5)	
	Emergency	34 (37.4)	9 (37.5)	25 (37.3)	
Primary diagnosis					
	Valve-related cardiac disease	30 (33.0)	7 (29.2)	23 (34.3)	0.835
	Aortic aneurysm	15 (16.5)	6 (25.0)	9 (13.4)	0.322
	Aortic dissection	13 (14.3)	6 (25.0)	7 (10.4)	0.159
	Ischemic heart disease	26 (28.6)	5 (20.8)	21 (31.3)	0.475
	Thromboembolic Pulmonary disease	4 (4.4)	0 (0.0)	4 (6.0)	0.520
	Cardiac tumor	3 (3.3)	0 (0.0)	3 (4.5)	0.698
Type of surgery					0.127
	Graft Replacement for aortic disease	28 (30.8)	12 (50.0)	16 (23.9)	
	Valve surgery	24 (26.4)	4 (16.7)	20 (29.9)	
	AMI complication repair	15 (16.5)	2 (8.3)	13 (19.4)	
	Coronary surgery	10 (11.0)	3 (12.5)	7 (10.4)	
	Coronary and Valve surgery	7 (7.7)	3 (12.5)	4 (6.0)	
	Pulmonary thrombectomy	4 (4.4)	0 (0.0)	4 (6.0)	
	Tumor resection	3 (3.3)	0 (0.0)	3 (4.5)	
EuroSCORE II		11.6 [4.9, 25.7]	14.8 [6.7, 42.1]	9.9 [3.7, 21.4]	0.081
Operation time, min		518 [354, 634]	604 [488, 707]	503 [314, 598]	0.012
Cardiopulmonary bypass time, min		258 [176, 362]	332 [240, 420]	245 [170, 323]	0.009
Arrest time, min		141 [87, 176]	156 [117, 192]	127 [85 172]	0.214
Unplanned additional CABG		20 (22.0)	6 (25.0)	14 (20.9)	0.897

Data are presented as medians [interquartile ranges] or numbers (%). AKI, acute kidney injury; AMI, Acute myocardial infarction; CABG, coronary artery bypass grafting; EuroScore, European system for cardiac operative risk evaluation; LVEF, left ventricular ejection fraction; NYHA, New York heart association.

When comparing the rapid-onset AKI group with the non-rapid-onset group, the median age was similar between the two groups (74.0 years [IQR, 62.5–80.2] vs. 71.0 years [IQR, 57.0–8.5], *p* = 0.430), and there were no significant differences in primary diagnosis or type of surgery. Preoperative serum creatinine levels (1.08 mg/dl [IQR, 0.81–1.39] vs. 0.95 mg/dl[IQR, [0.78–1.21], *p* = 0.222) and estimated glomerular filtration rate (eGFR; 44.0 ml/min/1.73 m^2^ [IQR, 29.3–63.2] vs. 50.0 ml/min/1.73 m^2^ [IQR, 37.5–65.2], *p* = 0.242) were also comparable. However, LVEF was significantly lower in the rapid-onset AKI group (42.5% [IQR, 28.8–58.5] vs. 60.0% [IQR, 45.0–65.0], *p* = 0.006).

For the overall cohort, the median durations of surgery, CPB, and cardiac arrest were 518 min [IQR, 354–634], 258 min [IQR, 176–362], and 141 min [IQR, 87–176], respectively. Unplanned intraoperative additional coronary artery bypass grafting was conducted in 20 cases (22.0%). The primary indications for V-A ECMO included failure to wean from CPB in 42 cases (46.2%), post-CPB heart failure in 34 cases (37.4%), and arrhythmias in 15 cases (16.5%). All arrhythmias occurred following failure to wean from cardiopulmonary bypass and were associated with cardiac arrest. These events were managed in the operating room, where all cardiac arrests were promptly treated with the initiation of V-A ECMO.

Peripheral V-A ECMO was employed in 76 patients (83.5%). 45 patients (49.5%) did not undergo any LV unloading. In comparison, 39 patients (42.9%) were supported with an IABP, two patients (2.2%) with an Impella device, and another five patients (5.5%) with left atrial (LA) or LV unloading. V-A ECMO was used as the primary modality of mechanical circulatory support in all patients. No patients were treated with IABP or Impella alone prior to V-A ECMO initiation.

When comparing the two groups, the rapid-onset AKI group showed longer operative time (604 min [IQR, 488–707] vs. 503 min[IQR, 315–598], *p* = 0.012) and CPB time (332 min [IQR, 240–420] vs. 245 min [IQR, 170–323], *p* = 0.009).

### 3.2 Postoperative clinical outcomes

The results at ICU admission and 24 h after ICU admission are presented in Table 2. The V-A ECMO flow index at ICU admission was 2.1 L/min/m^2^ [IQR, 1.7–2.5], with 42 patients (46.2%) receiving a V-A ECMO flow index greater than 2.4 L/min/m^2^. RRT was initiated within 24 h after surgery for four patients (4.4%) and at 24 h after ICU admission. The median postoperative LVEF at 24 h after ICU admission was 35.0 [IQR, 25.0–45.0], and 43 patients (47.3%) had an LVEF of 30% or less.

**Table 2:** Early postoperative results: types of PCCS and hemodynamic parameters.

Variable	Category	Overall	Rapid-onset postoperative AKI	No rapid-onset postoperative AKI	*p* value
*n*		91	24	67	
Reasons for ECMO					0.327
	Unable to wean CPB	42 (46.2)	9 (37.5)	33 (49.3)	
	HF after CPB	34 (37.4)	12 (50.0)	22 (32.8)	
	Arrythmia	15 (16.5)	3 (12.5)	12 (17.9)	
Type of heart failure					0.256
	LV failure	51 (56.0)	12 (50.0)	39 (58.2)	
	RV failure	13 (14.3)	3 (12.5)	10 (14.9)	
	Biventricular failure	12 (13.2)	6 (25.0)	6 (9.0)	
VIS Score Just Before ECMO Initiation		21.0 [9.5, 35.0]	31.9 [13.0, 59.0]	17.0 [8.0, 31.2]	0.028
VIS score >24 just before ECMO initiation		42 (46.2)	15 (62.5)	27 (40.3)	0.102
ECMO selection					0.103
	Peripheral ECMO	76 (83.5)	17 (70.8)	59 (88.1)	
	Central ECMO	15 (16.5)	7 (29.2)	8 (11.9)	
Primary LV unloading device					0.019
	None	45 (49.5)	14 (58.3)	31 (46.3)	
	IABP	39 (42.9)	7 (29.2)	32 (47.8)	
	Impella	2 (2.2)	2 (8.3)	0 (0.0)	
	LA drainage	1 (1.1)	1 (4.2)	0 (0.0)	
	LV drainage	4 (4.4)	0 (0.0)	4 (6.0)	
ECMO parameters					
At ICU admission	ECMO flow, L/min	3.5 [2.6, 4.1]	3.1 [2.4, 4.3]	3.5 [3.0, 4.0]	0.586
	ECMO flow index, L/min/m2	2.1 [1.7, 2.5]	2.1 [1.5, 2.4]	2.2 [1.7, 2.5]	0.402
At 24 h after ICU admission	ECMO flow, L/min	3.4 [2.5, 4.1]	3.5 [2.4, 3.8]	3.3 [2.5, 4.2]	0.850
	ECMO flow index, L/min/m2	2.0 [1.6, 2.5]	2.0 [1.8, 2.2]	2.0 [1.6, 2.5]	0.931
Hemodynamic parameters					
At ICU admission	Mixed venous oxygen saturation	81.2 [75.0, 87.0]	81.2 [75.7, 85.3]	81.2 [74.2, 87.2]	0.736
	Lactate level, mmol/L	5.3 [3.5, 7.5]	5.6 [4.0, 7.5]	5.3 [3.5, 7.3]	0.753
	hemoglobin, g/dl	9.9 [9.2, 10.8]	9.7 [8.7, 11.0]	9.9 [9.3, 10.8]	0.463
	VIS score	8.0 [5.0, 18.4]	12.3 [7.1, 27.2]	6.0 [4.0, 13.2]	0.007
At 24 h after ICU admission	Mixed venous oxygen saturation, %	78.1 [73.0, 84.0]	79.0 [71.0, 87.0]	78.0 [73.8, 82.2]	0.668
	Lactate level	2.4 [1.6, 3.5]	3.3 [1.8, 4.3]	2.0 [1.6, 3.3]	0.020
	Hemoglobin	10.6 [10.0, 11.0]	10.5 [9.9, 10.8]	10.6 [10.1, 11.1]	0.087
	VIS score	8.8 [4.9, 15.0]	8.7 [5.2, 15.1]	9.8 [4.3, 14.6]	0.528
LVEF at 24 h after ICU admission		35.0 [25.0, 45.0]	25.0 [25.0, 35.0]	35.0 [25.0, 45.0]	0.007
LVEF < 30% at 24 h after ICU admission		43 (47.3)	16 (66.7)	27 (40.3)	0.047
Total Bilirubin	At ICU admission	1.7 [1.1, 2.2]	1.8 [1.1, 2.3]	1.7 [1.2, 2.1]	0.853
	At 24 h after ICU admission	2.5 [2.0, 3.7]	2.3 [2.0, 3.7]	2.5 [2.0, 3.7]	0.586

Data are presented as medians [interquartile ranges] or numbers (%). AKI, acute kidney injury; CPB, cardiopulmonary bypass; ECMO, extracorporeal membrane oxygenation; HF, heart failure; ICU, intensive care unit; LA, left atrium; LV, left ventricle; LVEF, left ventricular ejection fraction; RV, right ventricle; VIS, vasoactive inotropic score.

The two groups showed no significant differences in V-A ECMO flow, flow index, mixed venous oxygen saturation, or lactate levels at ICU admission. However, the VIS was higher in the rapid-onset AKI group (12.3 [IQR, 7.1–27.2] vs. 6.0 [IQR, 4.0–13.2], *p* = 0.007). At 24 h after ICU admission, lactate levels were significantly elevated in the rapid-onset AKI group (3.3 mmol/L [IQR, 1.8–4.3] vs. 2.0 mmol/L [IQR, 1.6–3.3], *p* = 0.02).

A total of nine patients (15.8%) were classified as Stage 2 and fifteen (26.3%) as Stage 3 AKI within 24 h postoperatively and were therefore categorized into the rapid-onset AKI group (Table 3). Ten patients (11.0%) were upgraded to an Ex-VAD and four (4.4%) received Impella in a delayed fashion within one week after the initiation of V-A ECMO. The weaning process from mechanical circulatory support (MCS), including V-A ECMO, Impella, and Ex-VAD, was completed in 78 patients (85.7%). Of these, 67 patients (85.9%) met the criteria for successful weaning, defined as survival for at least 30 days following device removal. In addition, two patients were transitioned to a durable LVAD. The median duration of MCS was 3.0 days [IQR, 2.0–7.0].

**Table 3:** Clinical outcome.

Variable	Category	Overall	Rapid-onset postoperative AKI	No rapid-onset postoperative AKI	*p* value
*n*		91	24	67	
Incidence of AKI at 24 h after ICU admission		57 (62.6)	24 (100.0)	33 (49.3)	<0.001
AKI stage at 24 h after ICU admission					<0.001
	Stage 1	33 (57.9)	0 (0.0)	33 (100.0)	
	Stage 2	9 (15.8)	9 (37.5)	0 (0.0)	
	Stage 3	15 (26.3)	15 (62.5)	0 (0.0)	
Initiation of RRT within 24 h		4 (4.4)	4 (16.7)	0 (0.0)	0.005
Total Urine Output in the First 24 h		2,270 [1,244, 3,606]	621 [244, 1,410]	2,917 [1,956, 4,193]	<0.001
Urine output, ml/kg/hr.		1.6 [0.8, 2.6]	0.4 [0.2, 0.8]	1.9 [1.3, 2.9]	<0.001
Use of Lasix		28 (30.8)	4 (16.7)	24 (35.8)	0.137
Overall initiation of RRT		32 (35.2)	20 (83.3)	12 (17.9)	<0.001
ECMO outcomes					
Up-grade to Ex-VAD		10 (11.0)	5 (20.8)	5 (7.5)	0.157
Add Impella		4 (4.4)	2 (8.3)	2 (3.0)	0.606
MCS weaning (ECMO/Impella/Ex-VAD)		78 (85.7)	18 (75.0)	60 (89.6)	0.159
Successful weaning		67 (85.9)	11 (61.1)	56 (93.3)	0.002
Conversion to durable LVAD		2 (2.2)	0 (0.0)	2 (3.0)	0.964
Total MCS support duration, days		3.0 [2.0, 7.0]	6.0 [3.0, 20.2]	2.0 [1.5, 5.5]	0.001
Incidence of complication on MCS support		19 (20.9)	10 (41.7)	9 (13.4)	0.009
Type of complication	Neurological complications	8 (8.8)	4 (16.7)	4 (6.0)	0.243
	Hemorrhage	6 (6.6)	5 (20.8)	1 (1.5)	0.005
	Vascular injury	2 (2.2)	1 (4.2)	1 (1.5)	1.000
	LA thrombosis	3 (3.3)	0 (0.0)	3 (4.5)	0.698
In-hospital mortality		30 (33.0)	17 (70.8)	13 (19.4)	<0.001
Mortality on MCS		11 (12.1)	6 (25.0)	5 (7.5)	0.058
Mortality on MCS or death within 30 days after decannulation		22 (24.2)	13 (54.2)	9 (13.4)	<0.001
90-days mortality		31 (34.1)	17 (70.8)	14 (20.9)	<0.001
Cause of Mortality	Multiple Organ Dysfunction Syndrome	15 (16.5)	7 (29.2)	8 (11.9)	0.103
	Sepsis	6 (6.6)	3 (12.5)	3 (4.5)	0.379
	Hemorrhagic event	4 (4.4)	4 (16.7)	0 (0.0)	0.005
	Neurological event	5 (5.5)	2 (8.3)	3 (4.5)	0.850
	Other event	1 (1.1)	1 (4.2)	0 (0.0)	0.590

Data are presented as medians [interquartile ranges] or numbers. AKI, Acute kidney injury; ECMO, Extracorporeal membrane oxygenation; ex-VAD, Extracorporeal ventricular assist device; LVAD, Left ventricular assist device; MCS, Mechanical circulatory support; RRT, Renal replacement therapy.

The success rate of MCS weaning was lower in the rapid-onset AKI group compared to the non-rapid-onset group (61.1% vs. 93.3%, *p* = 0.002), and the duration of MCS support was significantly longer (6.0 days vs. 2.0 days, *p* = 0.001). Complications during MCS support occurred in 19 patients (20.9%).

Ninety-day mortality was observed in 31 patients (34.1%) overall and was significantly higher in the rapid-onset AKI group (70.8% vs. 20.9%, *p* < 0.001).

During MCS management, bleeding events were more frequent in the rapid-onset AKI group (20.8% vs. 1.5%, *p* = 0.005), and the mortality rate during MCS support or within 30 days after weaning from MCS was higher (54.2% vs. 13.4%, *p* = 0.058). The most common cause of death was multiorgan failure (29.2% vs. 11.9%, *p* = 0.103), followed by bleeding-related events (16.7% vs. 0%, *p* = 0.005).

### 3.3 Survival analysis

**Figure 2:**
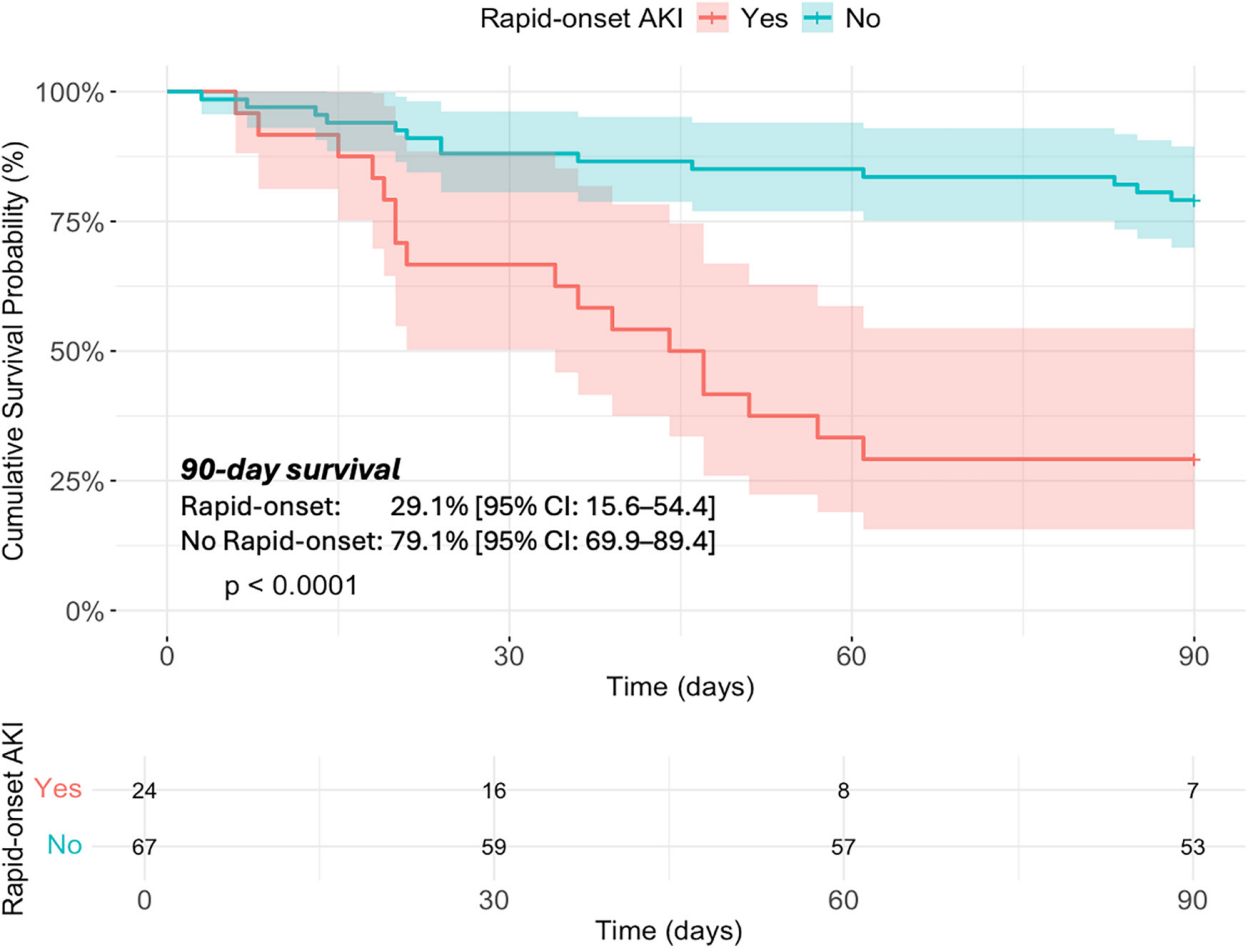
90-day survival in patients with post-cardiotomy cardiogenic shock stratified by incidence of rapid-onset AKI.

Kaplan–Meier analysis showed a significantly lower 90-day cumulative survival probability in the rapid-onset AKI group (29.1% [95% confidence interval (CI): 15.6–54.4]) compared to the non-rapid-onset AKI group (79.1% [95% CI: 69.9–89.4], *p* < 0.001) (Figure 2). Multivariate Cox regression analysis identified the occurrence of rapid-onset AKI (adjusted hazard ratio [aHR]: 3.15, 95% CI: 1.38–7.19, *p* = 0.006) (Table 4). Other factors associated with 90-day mortality included age (per 10-year increase, adjusted hazard ratio [aHR]: 1.32, 95% CI: 1.00–1.73, *p* = 0.048), a primary diagnosis of aortic dissection (aHR: 2.72, 95% CI: 1.18–6.27, *p* = 0.019), and postoperative LVEF (per 10% increase, aHR: 0.49, 95% CI: 0.30–0.79, *p* = 0.004).

**Table 4:** Cox multivariate analysis between rapid-onset postoperative kidney injury and 90-day mortality.

Variable	Hazard ratio (95% CI)	*p* value
Incidence of Rapid-onset postoperative AKI	**3.15** (**1.38, 7.19)**	**0**.**006**
Age (per 10 years)	**1.32** (**1.00, 1.73)**	**0**.**048**
Primary diagnosis of aortic dissection	**2.72** (**1.18, 6.27)**	**0**.**019**
Cardiopulmonary bypass time (per 1 h)	1.05 (0.92, 1.21)	0.465
Lactate level at 24 h after ICU admission	1.16 (0.91, 1.48)	0.232
LVEF at 24 h postoperatively (for every 10% increase)	**0.49** (**0.30, 0.79)**	**0**.**004**

AKI, acute kidney injury; LVEF, left ventricular ejection fraction.

Bold values indicate statistical significance (*p* < 0.05).

## 4 Discussion

In our study, patients with rapid-onset AKI—defined as KDIGO Stage 2 or higher within 24 h of ICU admission—had lower preoperative cardiac function, longer CPB times, and required prolonged MCS compared to those without AKI. Although mortality during MCS support did not differ significantly between groups, the 30-day survival rate after weaning was significantly lower in the rapid-onset AKI group. Multivariate Cox analysis confirmed rapid-onset AKI as an independent predictor of 90-day mortality. The leading causes of death were multiorgan failure and bleeding.

It is well-known that increases in creatinine levels and the incidence of AKI after cardiac surgery negatively impact both short-term and long-term prognosis. Approximately 80% of patients undergoing V-A ECMO for cardiogenic shock develop AKI, with in-hospital mortality rates around 26% for AKI stage 1 and exceeding 60% for AKI stage 2 or higher. Moreover, reports suggest that combining the Society for Cardiovascular Angiography and Interventions (SCAI) shock stage with the AKI stage can effectively predict in-hospital mortality (14).

In this study, we focused on cases in which stage 2 or higher AKI developed within 24 h after cardiac surgery and identified an association with reduced 90-day survival. AKI occurring within this early postoperative window likely reflects systemic stress responses such as intraoperative and immediate postoperative hypoperfusion, ischemia-reperfusion injury, and acute inflammation (25). These processes suggest that Rapid-onset AKI represents not only a change in renal function but also an early indicator of systemic deterioration and multiorgan dysfunction.

Although the use of additional MCS devices, including Ex-VAD and Impella, was comparable between groups, the rapid-onset AKI group exhibited a significantly longer duration of MCS support and a lower successful weaning rate of only 61.1%. The combined effects of AKI-associated multiorgan failure, MCS-related hemorrhagic events, and postoperative low cardiac output may explain these findings. The presence or absence of AKI within 24 h postoperatively may serve as a valuable predictor of short-term outcomes and support early postoperative risk stratification. It may also provide a rationale for timely interventions, including enhanced bleeding risk management and organ failure prevention. Recent studies have proposed revising the definition of AKI specifically for cardiac surgery patients, suggesting that using a higher threshold for serum creatinine change (0.55 mg/dl) than the conventional criteria may improve the accuracy of prognostic prediction (26). This raises the possibility that, in the context of postcardiotomy syndrome (PCS), both the timing of AKI onset and its severity should be reconsidered to better reflect patient outcomes.

In the present study, postoperative cardiac dysfunction was also associated with poor prognosis. In this patient population, the use of MCS is intended not only to promote the recovery of multiple organ dysfunction but also to serve as a bridge to long-term therapies such as durable LVAD or heart transplantation. Our findings showed a high mortality rate following MCS weaning, suggesting that the ability to discontinue MCS does not necessarily indicate sufficient recovery from organ dysfunction. Moreover, this may imply that, in some patients, MCS did not adequately fulfill its role as a bridge-to-bridge (BTB) strategy.

When recovery from organ failure is inadequate at the time MCS weaning criteria are met, the decision to proceed with weaning often requires careful discussion among the clinical team. In elderly patients or those with MCS-related complications, weaning may be selected despite incomplete recovery, potentially leading to deterioration of organ function and subsequent death. This context may also explain why multiple organ failure and bleeding events were frequently identified as causes of death in our cohort.

Although early recovery from multiorgan failure is desirable and higher flow support might be beneficial, achieving this can be challenging in patients with PCCS, V-A ECMO may increase afterload, potentially leading to complications such as pulmonary congestion and left ventricular thrombus formation. Management strategies for such cases include using direct left ventricular venting methods, such as Impella or central V-A ECMO combined with LA/LV venting (27). However, these approaches carry risks of bleeding and hemolysis, which may further worsen AKI, requiring careful consideration (28–32). Furthermore, a unique aspect of clinical practice in Japan is that durable LVAD and heart transplantation are generally not indicated for patients aged 65 years or older, or for those with significant organ dysfunction. As a result, aggressive treatment, including the use of MCS as a bridge-to-bridge (BTB) strategy, may not be pursued in such cases. In our study, 63.7% of the patients were aged ≥65 years, and a significant number of deaths occurred within 30 days after MCS weaning, which may have influenced the overall outcomes. Nevertheless, the increased use of devices such as Impella in Japan since 2019 suggests that treatment strategies combining multiple MCS modalities will likely play an increasingly important role in improving survival rates.

Additionally, in patients with organ dysfunction such as AKI and those aged ≥65 years, durable LVADs are not indicated in Japan, and in some cases, aggressive treatment may not be pursued. In the present study, 63.7% of the patients were aged ≥65 years, and many deaths occurred within 30 days after MCS weaning, which likely influenced the outcomes. Nevertheless, with the increased use of devices such as Impella in Japan since 2019, treatment strategies that combine various MCS modalities are expected to become increasingly important in improving survival.

## 5 Limitations

This study has several limitations. First, as a single-center retrospective observational study, the generalizability of the findings is limited. It remains unclear whether similar trends would be observed in patient populations from other institutions or regions; therefore, further multicenter studies are needed to confirm the external validity of the results. Although multivariate analysis was used to adjust for confounding factors, the potential influence of unmeasured confounders cannot be ruled out. Additionally, the sample size of this study was limited, and due to its inherent nature, a comprehensive evaluation of all relevant factors was challenging. Thus, it is important to consider that unknown factors may also affect the prognosis. Furthermore, this study could not investigate the impact of rapid left ventricular unloading on prognosis. Given these limitations, careful interpretation of the study findings is warranted, and future multicenter collaborative studies and prospective research are needed to provide higher-quality evidence.

## 6 Conclusion

Rapid-onset AKI, defined as KDIGO Stage 2 or higher within 24 h of ICU admission, was significantly associated with increased 90-day mortality in patients with postcardiotomy cardiogenic shock requiring V-A ECMO.

## Data Availability

The raw data supporting the conclusions of this article will be made available by the authors, without undue reservation.
